# Supplementary material for: Intracellular trafficking of Notch orchestrates temporal dynamics of Notch activity in the fly brain
Source: Nat Commun. 2021 Apr 7;12:2083. doi: 10.1038/s41467-021-22442-3 (PMC8027629; doi:10.1038/s41467-021-22442-3)
Supplement: Supplementary file 1 — Supplementary Information [file 41467_2021_22442_MOESM1_ESM.pdf]

Supplementary Information

Intracellular trafficking of Notch orchestrates temporal dynamics of Notch activity in the fly brain

Wang et al.

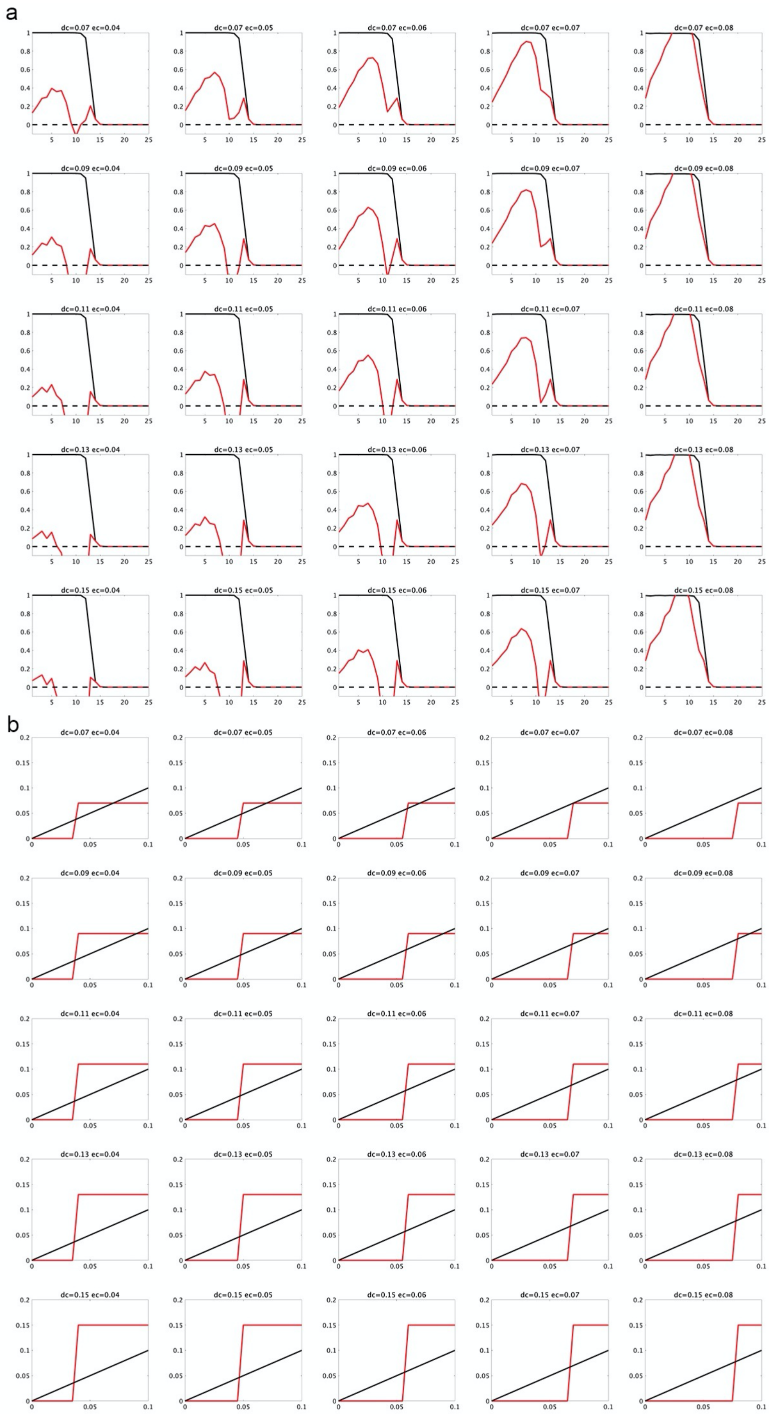

Supplementary Figure 1. Parameter range for step function in *cis*-inhibition that shows the twin peaks of Notch activity.

(a, b) The one-dimensional spatial patterns of N activity are compared by changing the values of magnitude (d_c) and threshold (e_c) for step function in *cis*-inhibition. (a) Profiles of N (red) and A (black). The value of N is 10 times multiplied. (b) Profiles of *trans*-activation (black) and *cis*-inhibition (red) in response to D. The values of *trans*-activation are four times multiplied because one cell could receive *trans*-activation from as many as four adjacent cells.

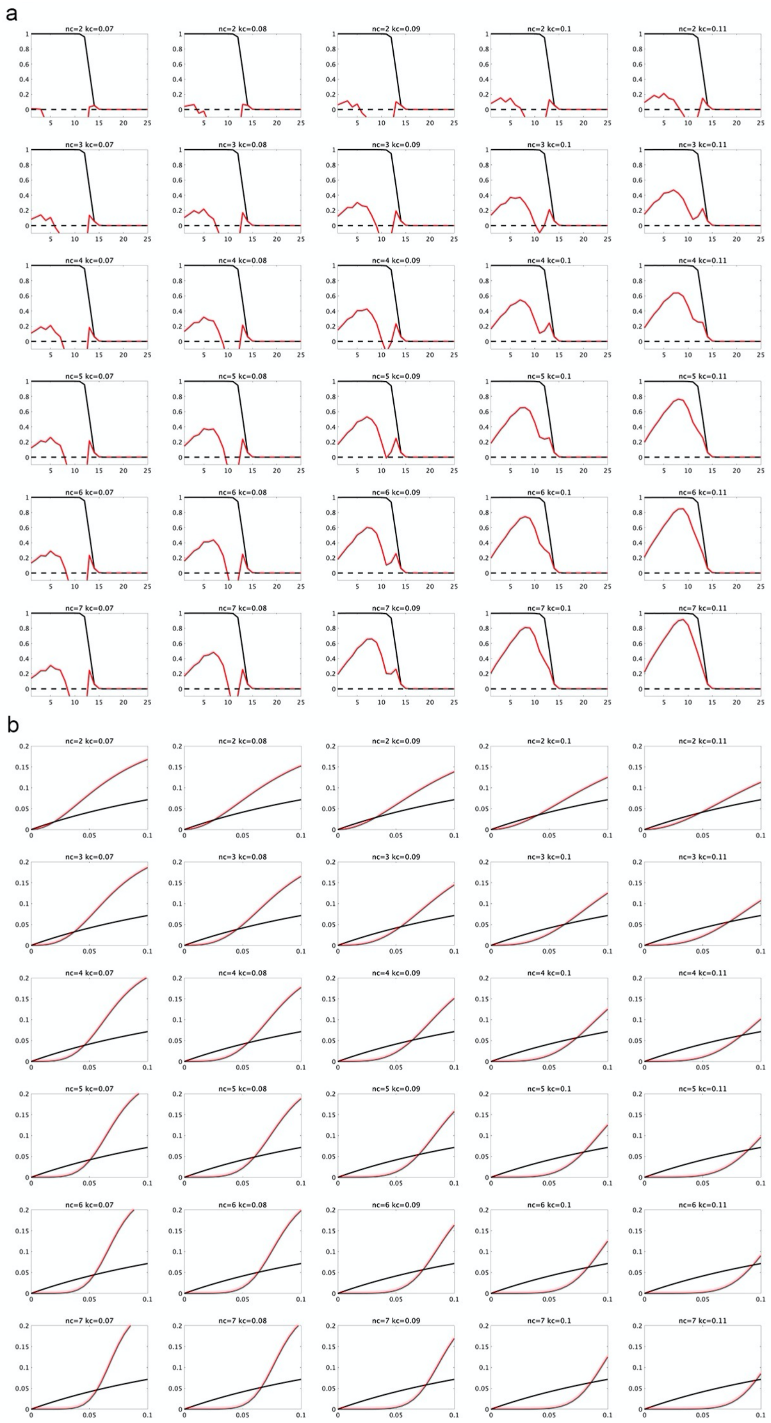

Supplementary Figure 2. Parameter range for Hill function in *cis*-inhibition that shows the twin peaks of Notch activity.

(a, b) The one-dimensional spatial patterns of N activity are compared by changing the values of Hill's co-efficient (n_c) and activation co-efficient (k_c) for Hill function in *cis*-inhibition. (a) Profiles of *N* (red) and *A* (black). The values of *N* is 10 times multiplied. (b) Profiles of *trans*-activation (black) and *cis*-inhibition (red) in response to *D*. The values of *trans*-activation are four times multiplied because one cell could receive *trans*-activation from as many as four adjacent cells.

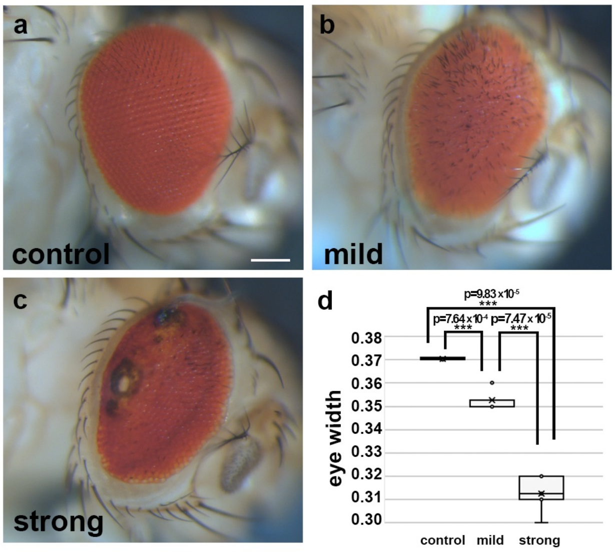

Supplementary Figure 3. Evaluating the strength of Delta RNAi strains.

(a, b, c) Control (a), mild *Dl* RNAi (b) and strong *Dl* RNAi (c) conditions under the control of *GMR-Gal4* show different eye size in adult at 17°C. Scale bar indicates 100um. (d) Quantification of eye size ($p=7.64x10^{-4}$, $9.83x10^{-5}$ and $7.47x10^{-5}$ for control/mild, control/strong and mild/strong, respectively (***:$p<0.001$), two-sided *t*-test, n=5, number of eye samples). Cross, mean; center line, median; box limits, upper and lower quartiles; whiskers, 1.5x interquartile range.

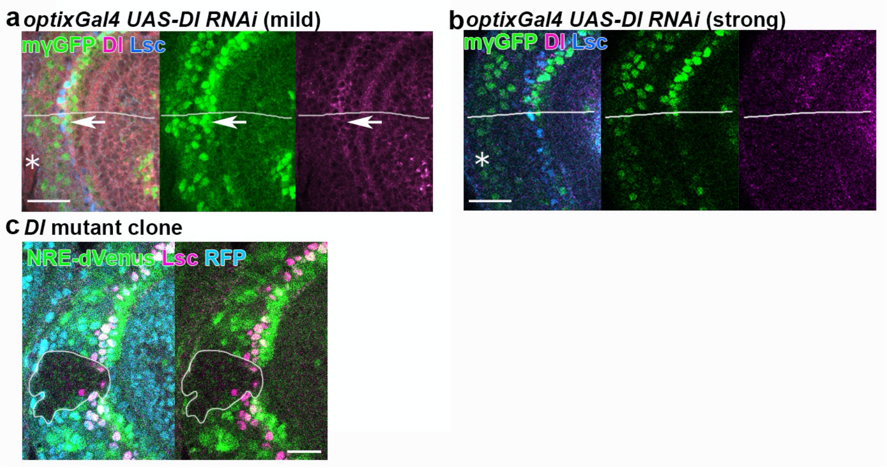

Supplementary Figure 4. Visualizing N activity with mγGFP in different *Dl* RNAi and *Dl* mutant conditions.

(a, b) Changes in N activity as visualized by mγGFP (green) upon *Dl* RNAi under the control of *optix-Gal4*. Dl (magenta) and Lsc (blue). (a) Partial knockdown of *Dl* causes the fusion of the twin peaks of N activity (arrows). (b) Strong knockdown of *Dl* causes the complete loss of Dl expression and N activity. Asterisks indicate the *optix-Gal4* positive areas outlined by white lines. (c) N activity visualized by NRE-dVenus (green) was lost in the *Dl* mutant clone visualized by the absence of RFP (blue). Scale bars indicate 20um.

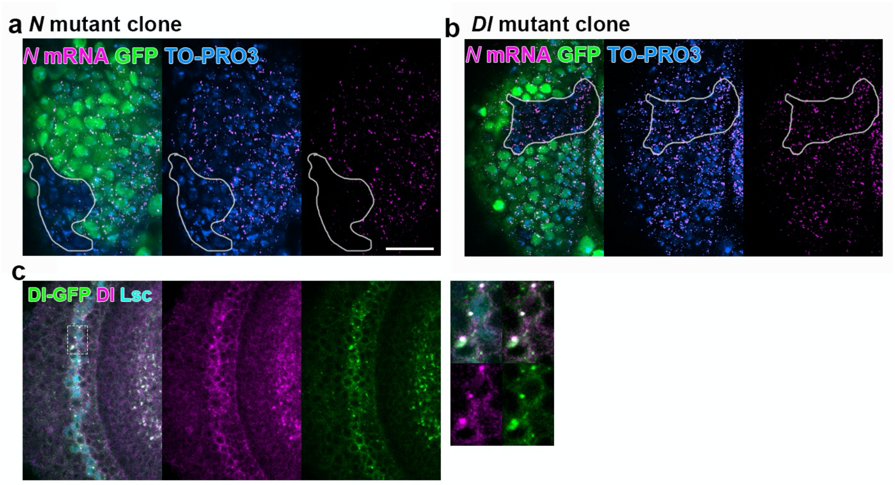

Supplementary Figure 5. ***N*** **mRNA distribution in** ***Dl*** **mutant clone.**

(a, b) *N* mRNA distribution was detected by using Stellaris RNA FISH Kit. (a) *N* mRNA (magenta) was lost in *N* mutant clones visualized by the absence of GFP (green). (b) *N* mRNA distribution was not affected in *Dl* mutant clone visualized by the absence of GFP (green). TO-PRO3 (blue). (c) Distribution patterns of Dl-GFP (green) and Dl antibody staining (magenta) at the wave front (Lsc; blue). White box is magnified in the right panel. Scale bar indicates 20um.

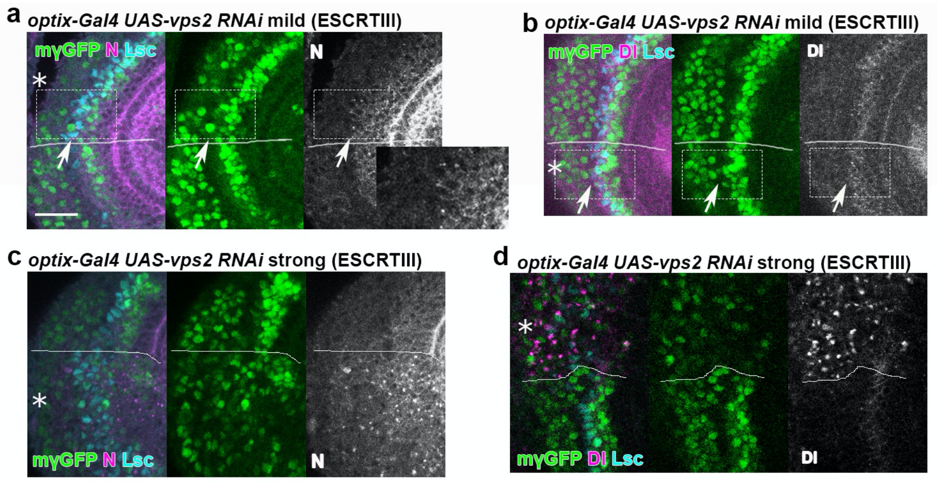

Supplementary Figure 6. Visualizing N activity with mγGFP in different *vps2* RNAi conditions.

(a, b) Fusion of the twin peaks of N activity as visualized by mγGFP (green) upon mild *vps2* RNAi under the control of *optix-Gal4* (arrows). N (magenta or white in a), Dl (magenta or white in b) and Lsc (blue). White boxes are magnified in the right panel showing the ectopic puncta of N (a). Dl expression is not significantly changed (b). (c, d) Broad N activation as visualized by mγGFP (green) upon strong *vps2* RNAi under the control of *optix-Gal4*. N (magenta or white in c), Dl (magenta or white in d) and Lsc (blue). N (c) and Dl (d) are broadly upregulated. Asterisks indicate the *optix-Gal4* positive areas outlined by white lines. Scale bars indicate 20um.

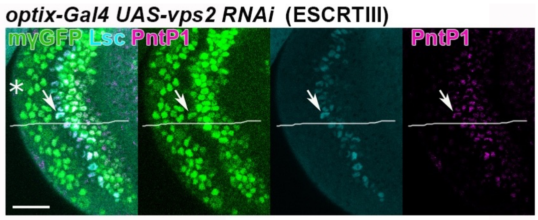

Supplementary Figure 7. ***vps2*** **RNAi does not significantly influence EGF activation.**
EGF signal activity as visualized by PntP1 (magenta) is not significantly altered by mild *vps2* RNAi under the control of *optix-Gal4* when the twin peaks of N activity are fused (mγGFP, green, arrows). Asterisk indicates the *optix-Gal4* positive areas outlined by white lines. Lsc, blue. Scale bars indicate 20um.

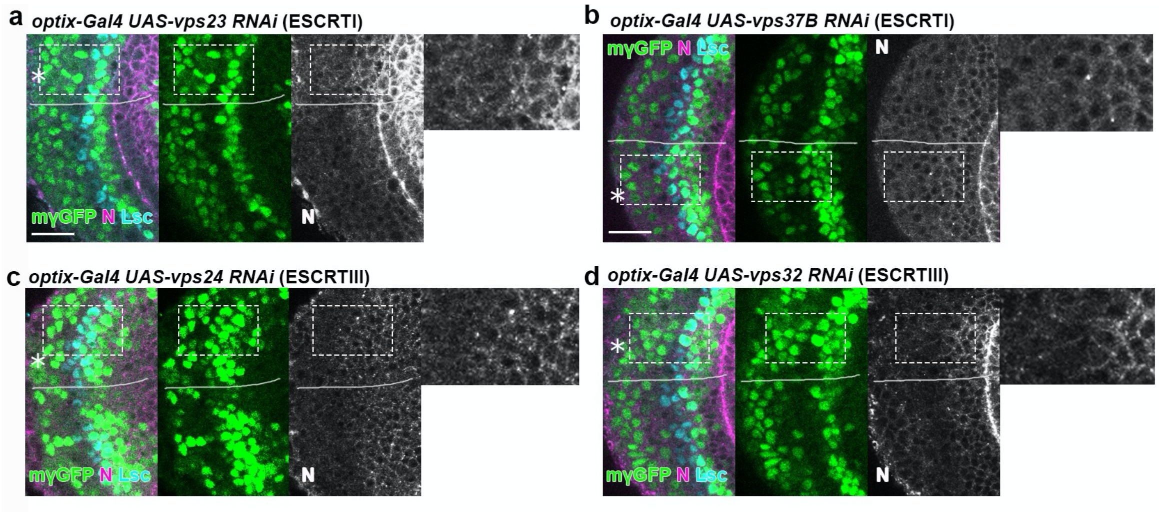

Supplementary Figure 8. ESCRT genes are involved in the formation of twin peaks of N activity.

RNAi for various ESCRT family genes (ESCRT I, II and III) under the control of *optix-Gal4* causes fusion of the twin peaks of N activity as visualized by mγGFP (green). N (magenta or white) and Lsc (blue). White boxes are magnified in the right panels showing the ectopic puncta of N. Asterisks indicate the *optix-Gal4* positive areas outlined by white lines. (a) *vps23* RNAi (ESCRT I). (b) *vps37* RNAi (ESCRT I). (c) *vps24* RNAi (ESCRT II). (d) *vps32* RNAi (ESCRT III). Scale bar indicates 20um.

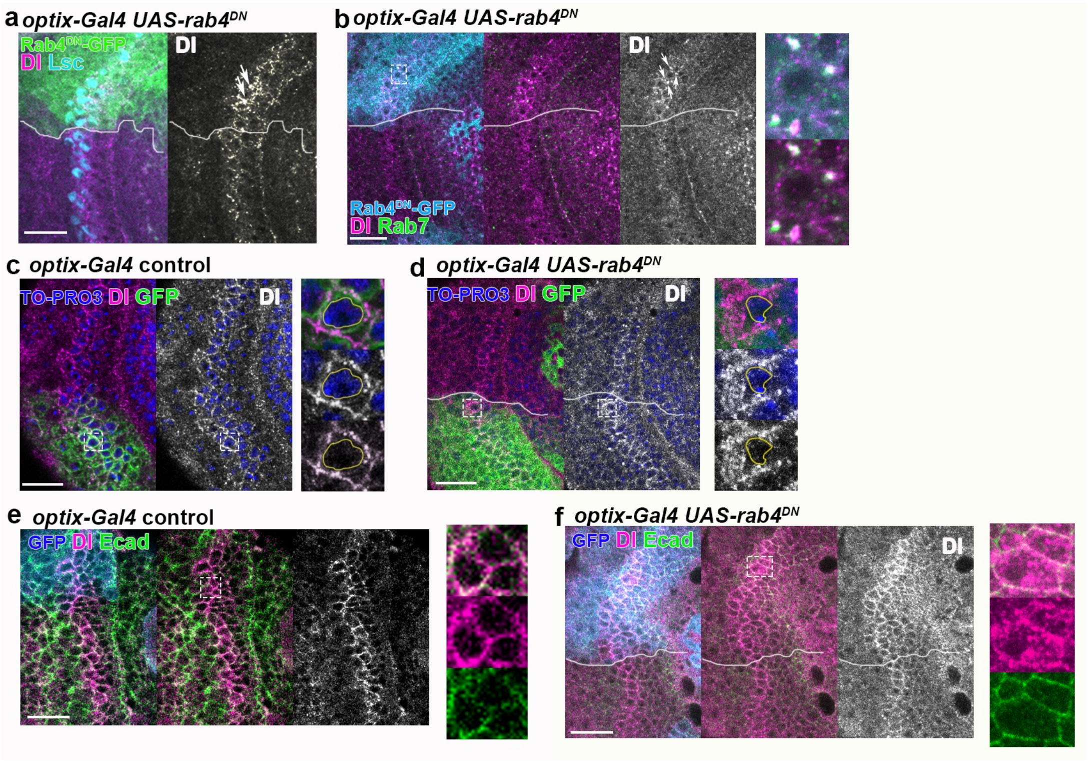

Supplementary Figure 9. Roles of recycling endosomes in Dl trafficking.

(a-f) Dl (magenta or white) is ectopically accumulated in the cytoplasm (arrows) upon *rab4*^{DN} expression under the control of *optix-Gal4* (GFP, green in a, c, d, blue in b) at the wavefront (Lsc, blue in a). Controls are shown in (c) and (e). Nuclei staining with TO-PRO3 (blue in c, d, outlined by yellow lines). Membrane staining with Ecad (green in e, f). The dotted boxes are magnified in the right panels. Scale bars indicate 20um.

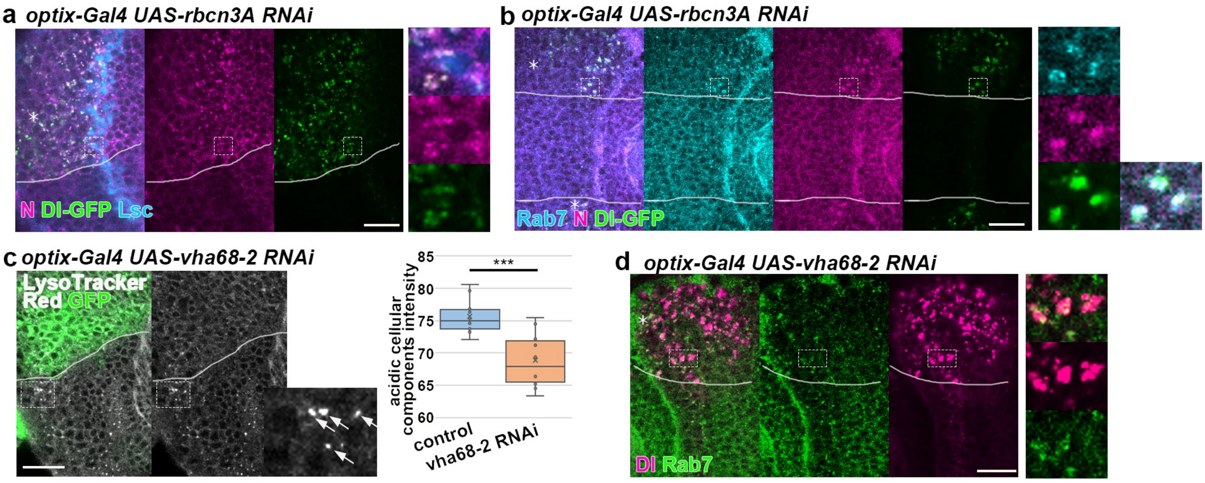

Supplementary Figure 10. Roles of Rbcn3A in Dl trafficking.

(a, b) N (magenta) and Dl-GFP (green) are ectopically accumulated and co-localized in the same puncta upon *rbcn3A* RNAi under the control of *optix-Gal4* behind the wavefront (Lsc, blue in a). The puncta co-localize with Rab7 (blue in b). (c) Acidic cellular components visualized by LysoTracker (white, arrows) are reduced by *vha68-2* RNAi under the control of *optix-Gal4* (GFP, green). Signal intensity is quantified ($p=0.0008$ (***: $p<0.001$), two-sided *t*-test, $n=10$, number of quantified areas). Cross, mean; center line, median; box limits, upper and lower quartiles; whiskers, 1.5x interquartile range. (d) Co-localization of Dl with Rab7 upon *vha68-2* RNAi under the control of *optix-Gal4* (control for Fig. 6m). Scale bars indicate 20um.

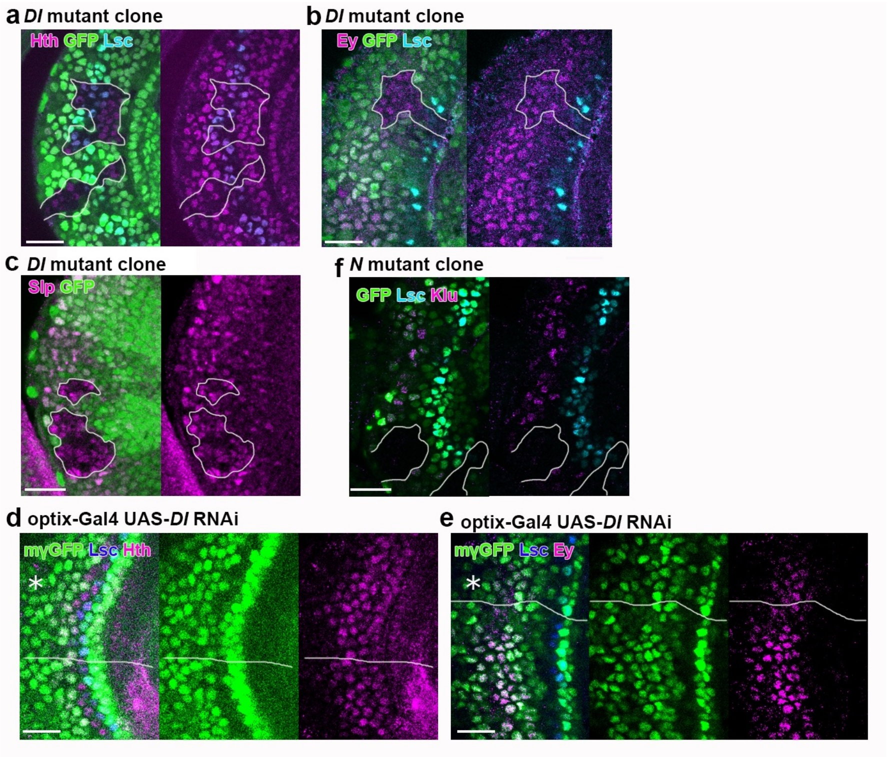

Supplementary Figure 11. Notch signaling regulates the temporal patterning of neurogenesis.

(a-c) Hth (magenta in a), Ey (magenta in b), and Slp (magenta in c) expression in *Dl* mutant clones visualized by the absence of GFP (green) behind the wavefront (Lsc, blue). (d, e) Fusion of the twin peaks of N activity as visualized by mγGFP (green) and expression of Hth (magenta in d) or Ey (magenta in e) upon partial knock down of *Dl*. (f) Klu (magenta) expression is eliminated in *N* mutant clones visualized by the absence of GFP (green). Lsc (blue). Scale bars indicate 20um.

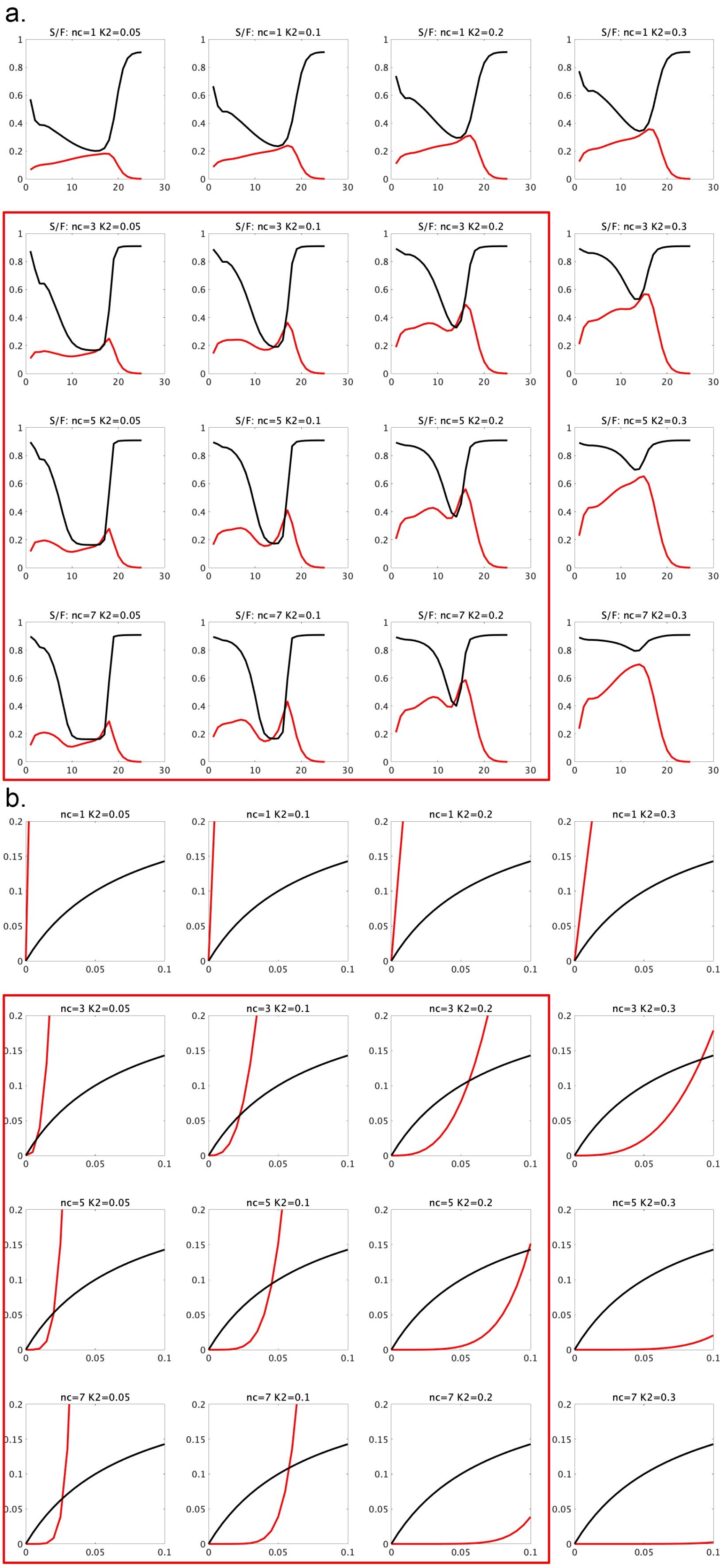
a.
S/F: nc=1 K2=0.05
S/F: nc=1 K2=0.1
S/F: nc=1 K2=0.2
S/F: nc=1 K2=0.3
S/F: nc=3 K2=0.05
S/F: nc=3 K2=0.1
S/F: nc=3 K2=0.2
S/F: nc=3 K2=0.3
S/F: nc=5 K2=0.05
S/F: nc=5 K2=0.1
S/F: nc=5 K2=0.2
S/F: nc=5 K2=0.3
S/F: nc=7 K2=0.05
S/F: nc=7 K2=0.1
S/F: nc=7 K2=0.2
S/F: nc=7 K2=0.3
b.
nc=1 K2=0.05
nc=1 K2=0.1
nc=1 K2=0.2
nc=1 K2=0.3
nc=3 K2=0.05
nc=3 K2=0.1
nc=3 K2=0.2
nc=3 K2=0.3
nc=5 K2=0.05
nc=5 K2=0.1
nc=5 K2=0.2
nc=5 K2=0.3
nc=7 K2=0.05
nc=7 K2=0.1
nc=7 K2=0.2
nc=7 K2=0.3

Supplementary Figure 12. Parameter range for *cis*-inhibition in the five-variable model that shows the twin peaks of Notch activity.

(a) The one-dimensional spatial patterns of N activity (S, red) and expression level of full-length N (F, black) are compared by changing the values of Hill's co-efficient (n_c) and activation co-efficient (K_2) for Hill function in *cis*-inhibition in the five-variable model. The value of S is 10 times multiplied. (b) Profiles of *trans*-activation (black) and *cis*-inhibition (red) in response to D. The values of *trans*-activation are four times multiplied because one cell could receive *trans*-activation from as many as four adjacent cells. The red framed panels show the twin peaks of Notch activity.
