# Supplementary material for: Intracellular trafficking of Notch orchestrates temporal dynamics of Notch activity in the fly brain
Source: Nat Commun. 2021 Apr 7;12:2083. doi: 10.1038/s41467-021-22442-3 (PMC8027629; doi:10.1038/s41467-021-22442-3)
Supplement: Supplementary file 3 — Reporting Summary [file 41467_2021_22442_MOESM3_ESM.pdf]

nature research

Corresponding author(s): Makoto Sato

Last updated by author(s): Mar 2, 2021

Reporting Summary

Nature Research wishes to improve the reproducibility of the work that we publish. This form provides structure for consistency and transparency in reporting. For further information on Nature Research policies, see our Editorial Policies and the Editorial Policy Checklist.

Statistics

For all statistical analyses, confirm that the following items are present in the figure legend, table legend, main text, or Methods section.

n/a	Confirmed	
☐	☒	The exact sample size (*n*) for each experimental group/condition, given as a discrete number and unit of measurement
☐	☒	A statement on whether measurements were taken from distinct samples or whether the same sample was measured repeatedly
☐	☒	The statistical test(s) used AND whether they are one- or two-sided<br>*Only common tests should be described solely by name; describe more complex techniques in the Methods section.*
☒	☐	A description of all covariates tested
☒	☐	A description of any assumptions or corrections, such as tests of normality and adjustment for multiple comparisons
☐	☒	A full description of the statistical parameters including central tendency (e.g. means) or other basic estimates (e.g. regression coefficient) AND variation (e.g. standard deviation) or associated estimates of uncertainty (e.g. confidence intervals)
☐	☒	For null hypothesis testing, the test statistic (e.g. *F*, *t*, *r*) with confidence intervals, effect sizes, degrees of freedom and *P* value noted<br>*Give P values as exact values whenever suitable.*
☒	☐	For Bayesian analysis, information on the choice of priors and Markov chain Monte Carlo settings
☒	☐	For hierarchical and complex designs, identification of the appropriate level for tests and full reporting of outcomes
☐	☒	Estimates of effect sizes (e.g. Cohen's *d*, Pearson's *r*), indicating how they were calculated

Our web collection on statistics for biologists contains articles on many of the points above.

Software and code

Policy information about availability of computer code

Data collection	ZEN 2.3
Data analysis	Fiji (ImageJ 1.53c), Adobe Photoshop v21.2, MATLAB R_2019b

For manuscripts utilizing custom algorithms or software that are central to the research but not yet described in published literature, software must be made available to editors and reviewers. We strongly encourage code deposition in a community repository (e.g. GitHub). See the Nature Research guidelines for submitting code & software for further information.

Data

Policy information about availability of data

All manuscripts must include a data availability statement. This statement should provide the following information, where applicable:

- Accession codes, unique identifiers, or web links for publicly available datasets
- A list of figures that have associated raw data
- A description of any restrictions on data availability

The authors declare that the data supporting the findings of this study are available within the paper.

Field-specific reporting

Life sciences study design

All studies must disclose on these points even when the disclosure is negative.

Sample size	At least ten samples were examined when available. Otherwise, samples size was determined so that the result shows a statistical significance.
Data exclusions	The damaged brain samples were always excluded from the analysis in all experiments. Otherwise, outliers or individual data were included in the graph.
Replication	All attempts of replication successfully reproduced the results.
Randomization	Samples were randomly collected from control and experimental groups.
Blinding	Blinding were not necessary because samples were randomly collected from control and experimental groups according to genetic labeling technique.

Reporting for specific materials, systems and methods

We require information from authors about some types of materials, experimental systems and methods used in many studies. Here, indicate whether each material, system or method listed is relevant to your study. If you are not sure if a list item applies to your research, read the appropriate section before selecting a response.

Materials & experimental systems

n/a	Involved in the study	
☐	☒	Antibodies
☒	☐	Eukaryotic cell lines
☒	☐	Palaeontology and archaeology
☐	☒	Animals and other organisms
☒	☐	Human research participants
☒	☐	Clinical data
☒	☐	Dual use research of concern

Methods

n/a	Involved in the study	
☒	☐	ChIP-seq
☒	☐	Flow cytometry
☒	☐	MRI-based neuroimaging

Antibodies

Antibodies used	guinea pig anti-Lsc (Makoto Sato, Kanazawa University, Japan), mouse anti-Dl (DSHB C594.9B), mouse anti-N (DSHB C17.9C6), rat anti-Ecad (DSHB DCAD2), rabbit anti-Klu (Xiaohang Yang, Singapore), guinea pig anti-Run (Asian Distribution Center for Segmentation Antibodies, Mishima, Japan), and rabbit anti-Rab7 antibodies (Akira Nakamura, Kumamoto University, Japan). anti-mouse Cy3 (Jackson ImmunoResearch 715-165-151), anti-mouse Cy5 (Jackson ImmunoResearch 715-175-151), anti-guinea pig Alexa647 (Jackson ImmunoResearch 712-605-150), anti-rabbit Alexa546 secondary antibodies (Invitrogen A-11035).
Validation	These antibodies were described and validated in published papers (Dev Biol 380, 12-24 (2013), Genes Dev 11, 1396-1408 (1997), Development 135, 1107-1117 (2008)), or were generated and validated by manufacturers (https://dshb.biology.uiowa.edu , https://shigen.nig.ac.jp/fly/nigfly/segmentationAntibodies/index.html , https://www.jacksonimmuno.com , https://www.thermofisher.com/antibody/product/Goat-anti-Rabbit-IgG-H-L-Highly-Cross-Adsorbed-Secondary-Antibody-Polyclonal/A-11035).

Animals and other organisms

Policy information about studies involving animals; ARRIVE guidelines recommended for reporting animal research

Laboratory animals	Drosophila melanogaster, Canton S strain, males and females of late third larval instar and 0 day old adult.
Wild animals	No wild animal was used in this study.
Field-collected samples	No field-collected sample was used in this study.
Ethics oversight	This study did not require an ethical approval.

Note that full information on the approval of the study protocol must also be provided in the manuscript.
